# Complex Hemifacial Degloving Injury to Face: A Rare Case Report

**DOI:** 10.29252/wjps.10.2.124

**Published:** 2021-05

**Authors:** Prashant Moon

**Affiliations:** 1 Krishna Hospital and Research Center, Gurunanak Pura, Haldwani, Uttarakhand 263139, India

**Keywords:** Degloving, Face, Reconstruction

## Abstract

Abrasions and laceration of face are very common injury in a road traffic accident. Complex Hemi-facial Degloving injury of face is very rare injury in road traffic accident. Reconstruction of face and rehabilitation of patient poses a great challenge to treating surgeon. Here a case of hemifacial Degloving injury of face in A 45-year-old female patient from India is reported.

## INTRODUCTION

Road traffic accidents often lead to multiple injuries to face. Common facial injuries after road traffic accidents are abrasion, laceration, maxillofacial trauma, and intraoral injuries ^1^. Other injuries like head injury, chest injury, and limb injuries are associated with facial injuries. Hemifacial Degloving injuries are very rare type of injury in a road traffic accident. Most of the patients are not even reaching to hospital after injury due to severe associated head injury. Here a case of hemifacial Degloving injury due to road traffic accident is presented ^2^^,^^3^. 

## CASE PRESENTATION

**Fig. 1:**
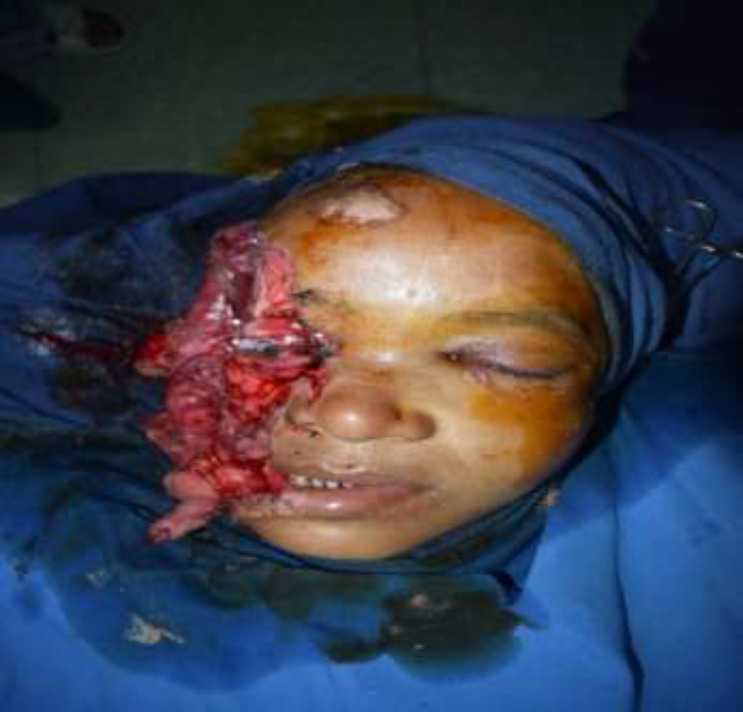
Preoperative picture

**Fig. 2:**
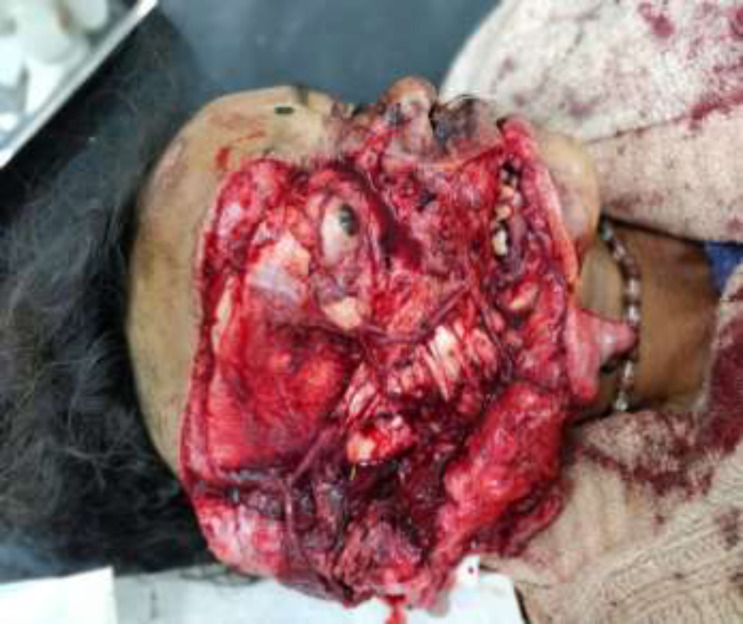
Preoperative picture lateral view

**Fig. 3:**
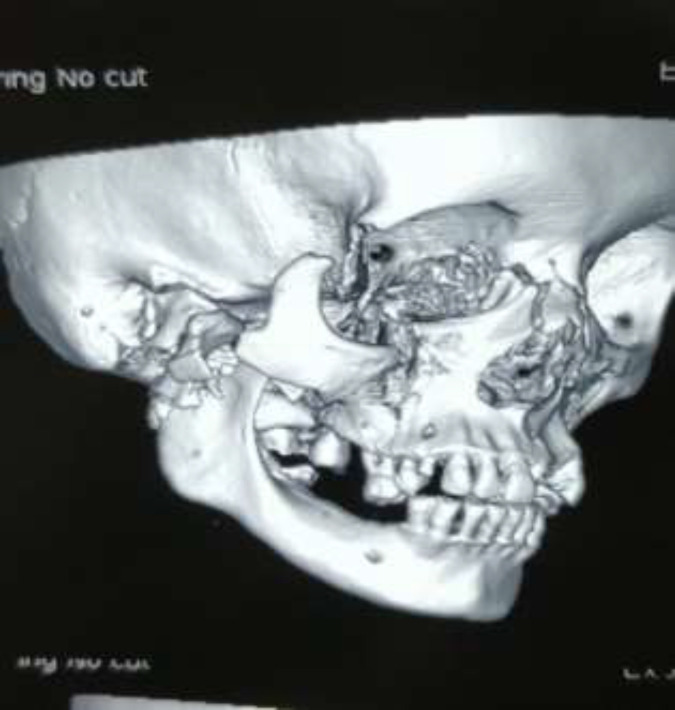
3D CT scan

A 45-year-old female patient was met with an accident due to collision between bike and truck. Female who was sitting on back seat of bike was fallen. Her face was caught in the rear tyre of a truck. The patient was immediately brought to hospital. The patient had profuse bleeding and was in shock. Initial resuscitation of patient was done in emergency department. A complete physical examination of patient was done. There was no obvious injury over her body except face. There was a hemifacial Degloving of right side of the face (Figure 1,2). Degloving of face was extending from temporal region of scalp, upper and lower conjunctiva, and cheek to upper and lower lip. Patient CT Scan of head and face done. NCCT head was normal. CT face suggestive of fracture of right condyle of mandible, right zygomatic arch comminuted fracture, fracture of right zygomatic-frontal buttress, right zygomatic-maxillary buttress, nasal bone fracture and fracture alveolar process of maxilla (Figure 3). Ophthalmology reference was done for ocular injury. There was corneal abrasion over

**Fig. 4:**
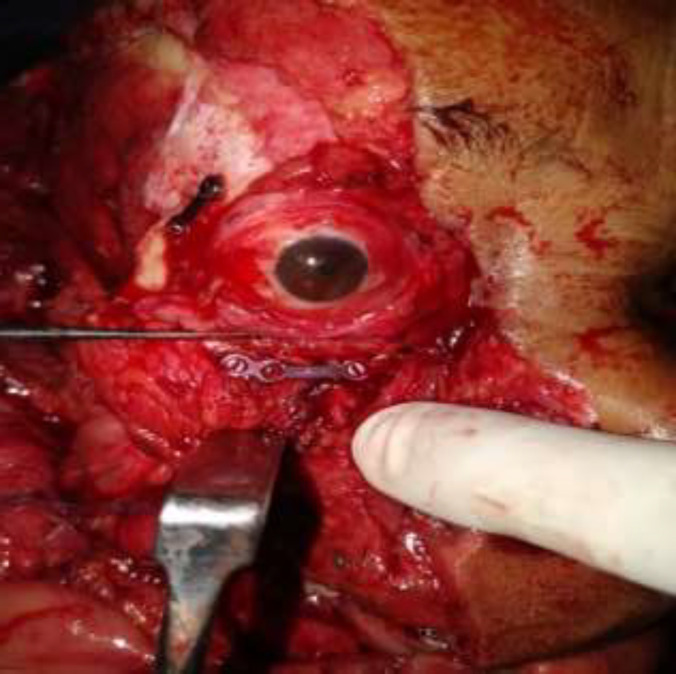
Fixation of fracture with titanium plate and screw

**Fig. 5:**
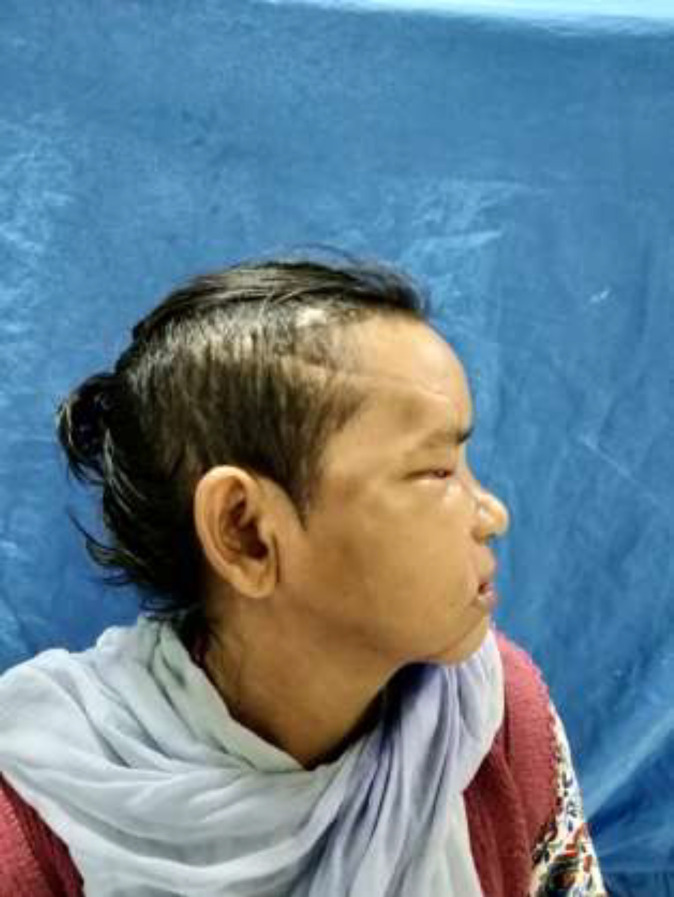
Post-operative image lateral view

**Fig. 6:**
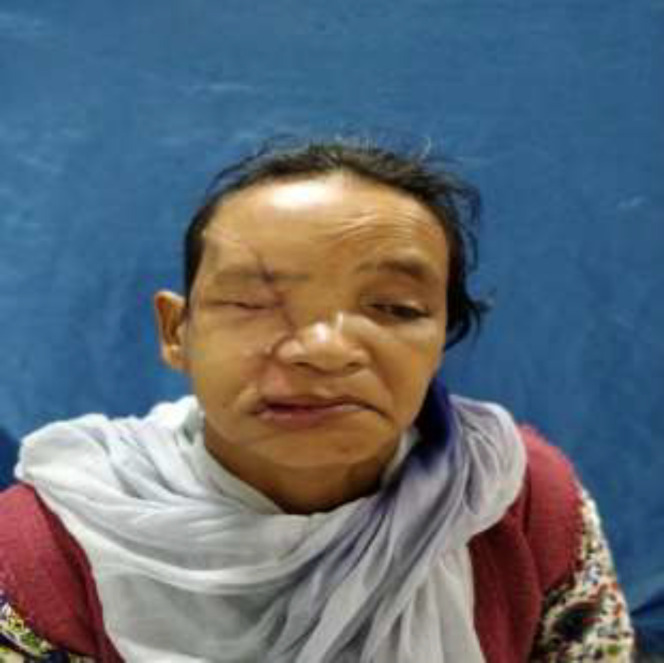
Post operative image frontal view

Routine investigations were sent and patient was shifted to OT for repair of Degloving injury. Nasal intubation was done. Thorough wash was given to remove dirt and dead tissue. Open reduction and closed fixation of right condyle of mandible and bilateral multiple zygomatic-maxillary bone fracture were done (Figure 4). Anatomical landmarks of avulsed hemifacial flap were identified and matched with its counterparts. Layered closure was performed to achieve oral competence. Left Upper and lower eyelid were avulsed from muco-cutaneus junction. Closure of muco-cutaneus junction was achieved. Closure of scalp wound was done over a suction drain. Dressing done and patient was shifted to intensive care unit for 2 days. Tarsorraphy of right eye was performed. Patient was discharged on postoperative day 9. Her post-operative recovery was uneventful except for right eye corneal scarring (Figure 5, 6).

Informed consent was taken from the patient. 

## DISCUSSION

Face has very complex anatomy. Due to the complexity of face, it is essential to anticipate the injuries in various structures underneath the wound. The first chance is the best chance for repair as it decides the outcome. Facial injury is not life-threatening unless it is associated with airway problems ^4^^, ^^5^. 

The major risks to the airway in patient's management with massive facial trauma are due to anatomic alteration of airway patency through bony disruption, soft tissue swelling and the increased potential for aspiration of body fluids ^5^. 

Immediate soft tissue reconstruction results in less scarring and infection. The secondary corrections are more complex and produce less satisfactory results than primary treatment ^6^.

The surgeon needs to understand the biomechanics of tissue wounding, biochemistry, molecular biology of wound healing and the art of soft-tissue repair. It is very important to identify important anatomical landmarks before repair ^6^. The first chance of repair is best chance as it decides the outcome. Anatomy of face is subdivided into various facial subunits. When there is a composite full-thickness loss of facial tissue, the requirement is lining, cover and support ^7^. Debridement is most important step in repair of facial injury. Handling of tissue should be atraumatic to minimize further tissue injury. 

Degloved tissue must be anchored to the fixed bony unit to avoid contractures. There should be special attention to important facial structures like eye, oral cavity, nose periorbital area, forehead and cheek. There should be repair of all layers of defect to achieve proper function and cosmesis ^8^. 

## CONCLUSION

Treatment is most successful if reconstructions are performed using well-vascularised tissues and respecting the aesthetic units of the face. Prognosis of vision is very poor in patient with upper and lower eyelid injury. 

## CONFLICT OF INTEREST

The author declares that there is no conflict of interests.
